# A ‘Comprehensive Visual Rating Scale’ for predicting progression to dementia in patients with mild cognitive impairment

**DOI:** 10.1371/journal.pone.0201852

**Published:** 2018-08-20

**Authors:** Jae-Won Jang, Jeong Hoon Park, Seongheon Kim, Young Ho Park, Jung-Min Pyun, Jae-Sung Lim, Youngho Kim, Young Chul Youn, SangYun Kim, the Alzheimer’s Disease Neuroimaging Initiative

**Affiliations:** 1 Department of Neurology, Kangwon National University Hospital, Kangwon National University College of Medicine, Chuncheon, Republic of Korea; 2 Department of Neurology, Seoul National University Bundang Hospital, Seongnam, Republic of Korea; 3 Department of Neurology, Seoul National University College of Medicine, Seoul, Republic of Korea; 4 Department of Neurology, Hallym University Sacred Heart Hospital, Hallym University College of Medicine, Anyang, Republic of Korea; 5 Department of Biomedical Engineering, Seoul National University Hospital, Seoul, Republic of Korea; 6 Department of Neurology, Chung-Ang University Hospital, Chung-Ang University College of Medicine, Seoul, Republic of Korea; University of New South Wales, AUSTRALIA

## Abstract

**Background:**

Numerous efforts have been made to identify biomarkers for predicting the progression of dementia in patients with mild cognitive impairment (MCI), and recently, a comprehensive visual rating scale (CVRS) based on magnetic resonance imaging (MRI) has been validated to assess structural changes in the brain of elderly patients. Based on this, the present study investigated the use of CVRS for predicting dementia and elucidated its association with cognitive change in patients with MCI over a three-year follow-up.

**Methods:**

We included 340 patients with MCI with more than one follow-up visit. Data were obtained from the Alzheimer’s disease Neuroimaging Initiative study. We assessed all the patients using CVRS and determined their progression to dementia during a follow-up period of over 3 years. The cox proportional hazards model was used to analyze hazard ratios (HRs) of CVRS for disease progression. Further, multiple cognitive measures of the patients over time were fitted using the random effect model to assess the effect of initial CVRS score on subsequent cognitive changes.

**Results:**

Of 340 patients, 69 (20.2%) progressed to dementia and the median baseline score (interquartile range) of CVRS significantly differed between stable MCI and progressive MCI (9 (5–13) vs 13 (8–17), p<0.001). The initial CVRS score was independently associated with an increased risk of progression to dementia (HR 1.123, 95% confidence interval [CI] 1.059–1.192). From 12 to 24 months, the effect of the interaction between CVRS and interval of follow-up visit on cognitive performance achieved significance (p<0.001).

**Conclusions:**

Baseline CVRS predicted the progression to dementia in patients with MCI, and was independently associated with longitudinal cognitive decline.

## Introduction

Mild cognitive impairment (MCI), a long predementia stage, is known to progress to dementia in approximately 15% of the patients annually[1]. This finding concurrently implies that approximately 85% of the patients with MCI remain clinically stable. Therefore, the need for risk assessment using biomarkers is imperative in patients with MCI to identify those with a high risk of progression to dementia [2].

Brain magnetic resonance imaging (MRI) is commonly used to assess individuals with cognitive decline and detect structural changes. The National Institute on Aging-Alzheimer’s Association (NIA-AA) adopted atrophy observed on structural MRI as neurodegenerative marker of Alzheimer’s disease (AD) in addition to increased CSF tau, hypometabolism on [18F]-fluorodeoxyglucose-PET, or positive tau PET[3–5]. However, AD-like atrophy primarily observed in temporal lobe occurs in a variety disorders, such as cerebrovascular disease, hippocampal sclerosis, TDP-43-opathy or primary age-related tauopathy[6,7]. Among these non-AD conditions that have been labeled as suspected non-Alzheimer pathophysiology (SNAP)[8], cerebrovascular lesion is one of the most common pathologic finding [9,10]. Considering that MCI is also frequently associated with multiple pathologies[11], it is necessary to develop neuroimaging markers that simultaneously reflect neurodegeneration and vascular injury.

To obtain a complete understanding of the structural changes due to atrophy and cerebrovascular lesions, a quantified comprehensive visual rating scale (CVRS) based on brain MRI has been developed[12]. The CVRS integrated the preexisting visual rating scales (hippocampal atrophy, cortical atrophy, ventricular enlargement, and small vessel disease) without losing the value of subscales[12]. Previously, CVRS was validated for individuals with normal cognition, MCI and, AD, and was found to reflect the structural changes observed in the brain of patients with MCI and AD, and significantly correlate with neuropsychological tests[12]. However, whether this scale can be used for predicting disease progression and its relationship with cognitive changes in longitudinal follow-ups is unclear. Hence, the current study aimed to investigate the use of CVRS for predicting progression to dementia over a 3-year follow-up period in the patients with MCI.

## Materials and methods

### Ethics statement

The institutional review board of Kangwon National University Hospital approved this study. The approval number is “KNUH-2017-04-012” and we did not have access to any identifying participant data. The study procedures were approved by the institutional review board of all participating centers (http://adni.loni.usc.edu/wp-content/uploads/how_to_apply/ADNI_Acknowledgement_List.pdf) and written informed consent was obtained from all participants or authorized representatives. Detailed protocols for informed consent of Alzheimer’s Disease Neuroimaging Initiative (ADNI) subjects can be referenced in ADNI information pages (www.adni-info.org.).

### Subjects

Data used in the preparation of this article were obtained from the Alzheimer’s Disease Neuroimaging Initiative (ADNI) database (adni.loni.usc.edu). The ADNI was launched in 2003 as a public-private partnership, led by Principal Investigator Michael W. Weiner, MD. The primary goal of ADNI has been to test whether serial MRI, PET, other biological markers, and clinical and neuropsychological assessments can be combined to measure the progression of MCI and early AD. For up-to-date information, see www.adni-info.org.

Data used in this study were downloaded from the ADNI database on the 21^th^ December, 2017. We included patients with MCI who had a baseline MRI scan as well as amyloid PET study, and at least one or more follow-up visits after initial assessment. The primary outcome of this study was progression to dementia during the follow-up period of up to 3 years. A final total of 340 patients from the ADNI-GO/ADNI2 cohort were included in this study.

Diagnosis of MCI was made according to the presence of objective memory impairment but without meeting the criteria for dementia. Namely, all subjects had a Mini Mental State Examination (MMSE) score of 24 or higher, a global Clinical Dementia Rating (CDR) score of 0.5, a CDR memory score of 0.5 or higher, and a score indicating impairment on the delayed recall of Story A of the Wechsler Memory Scale-Revised (≥16 years of education: ≤8; 8–15 years of education: ≤4; 0–7 years of education: ≤2)[13]. Diagnosis of dementia at follow-up was made according to the presence of memory complaints, a CDR score ≥0.5, and significant impairments on objective cognitive measures and in ADL. Individuals with AD met the National Institute of Neurological and Communicative Disorders and Stroke-Alzheimer’s Disease and Related Disorders Association criteria for probable AD[14].

### MRI

All subjects were imaged using a 3-T MRI scanner (GE, Siemens, or Philips). Data were collected at multiple ADNI sites in accordance with a standardized MRI protocol (http://adni.loni.usc.edu/methods/documents/mri-protocols/) that was developed by comparing and evaluating 3D T1-weighted sequences for morphometric analyses. MRI acquisition and processing were performed as per standard protocol[15]. Preprocessed T1-weighted MPRAGE MR images (T1-W MRI), a fluid-attenuated inversion recovery image (FLAIR), a T2 star weighted image were downloaded from the ADNI database.

### Comprehensive visual rating scale (CVRS)

**Fig 1:**
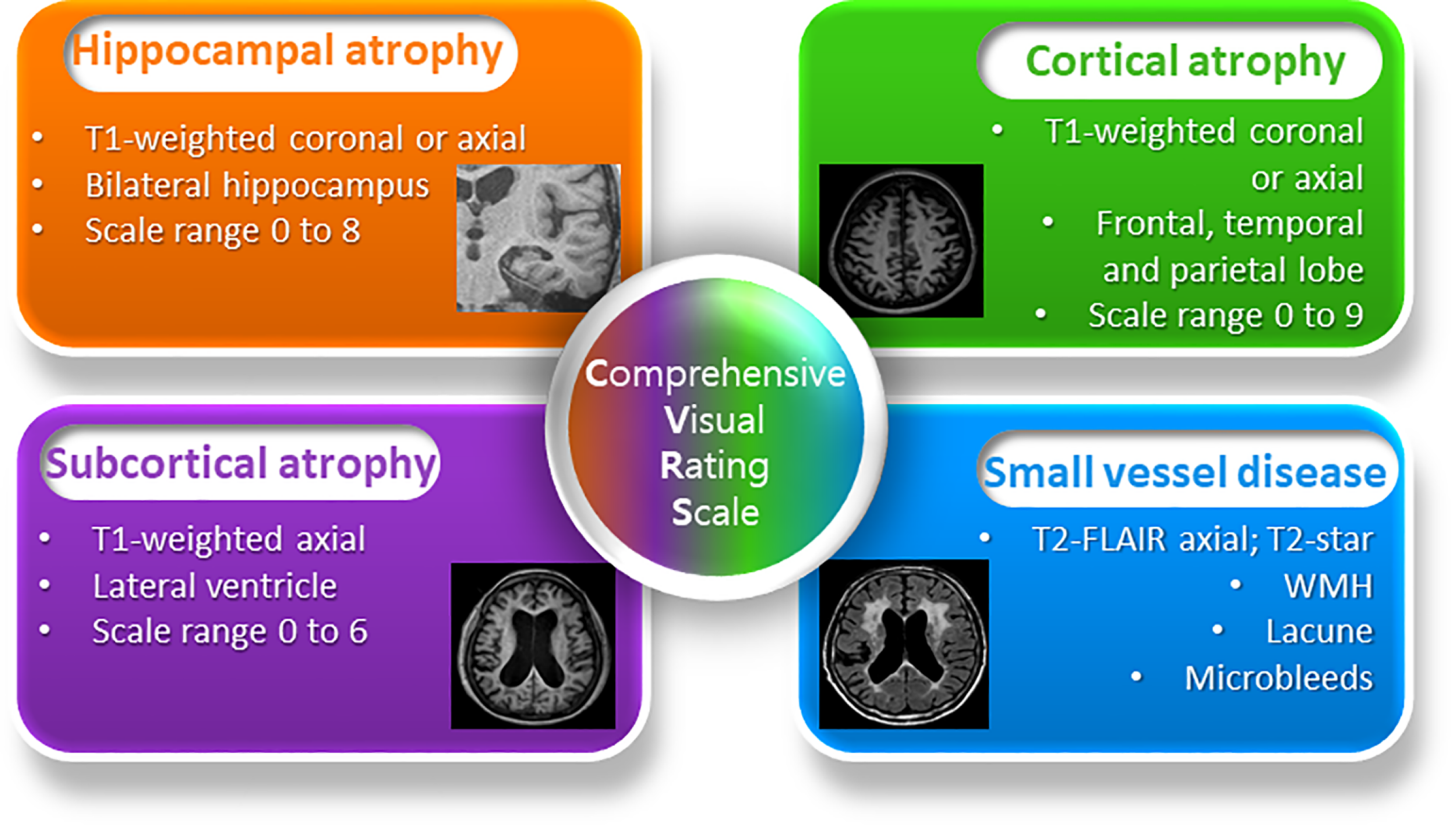
Construction of comprehensive visual rating scale(CVRS).

The CVRS includes the scales of hippocampal atrophy, cortical atrophy, ventricular enlargement (subcortical atrophy), and small vessel disease, which summarize degenerative or vascular injury of the aged brain (Fig 1, S1 Fig). The details of each scale are described elsewhere[12] and S1 File. The CVRS has adopted these existing scales that have been validated, and combined them to quantify the effects of multiple brain deficits, thus yielding a scale with scores ranging from 0 to 30 (a higher score represents more deficits).

The visual rating was performed by three raters (Jae-Won Jang, Jeong Hoon Park, Seongheon Kim), who were blind to demographic and clinical information. The rater used a template-based scoring program on a tablet computer that calculated the total score automatically by matching the closest template image to MRI findings of the patient (S2 File). The inter-rater and intra-rater reliability with 34 randomly selected MRI scans were 0.941 and 0.936 respectively (S1 Table). Cross-sectional validation of a clinical group including individuals with normal cognition, MCI, and dementia was performed in previous study[12].

### [^18^F]AV45 PET

The [^18^F]AV45 PET mean standard uptake value ratio (SUVR) was determined for each subject. Aβ-positive (Aβ+) and Aβ-negative (Aβ-) status were defined according to a SUVR threshold of ≥1.10. This threshold was taken from the ADNI database as the composite volume of interest (VOI) standardized uptake value ratio (SUVR) with the highest accuracy for discriminating between cognitively normal subjects and patients with AD[16].

### Neuropsychological data

Longitudinal neuropsychological markers such as MMSE score, Alzheimer’s Disease Scale Cognitive Subscale (ADAS-cog) [17] score, and Clinical Dementia Rating-Sum of Boxes (CDR-SOB) score were evaluated at baseline up to 3-years by one-year intervals.

### Statistical analysis

Independent t-tests and chi-square tests were used to examine between-group differences in continuous variables and categorical variables, respectively. Mann-Whitney U test was used for continuous variables that are not normally distributed. We assessed the hazard ratio (HR) of the CVRS, baseline demographics, and neuropsychological profiles using univariate Cox regression analysis with follow-up time as the time variable and progression to dementia as the status variable. Kaplan-Meier plots were used to determine whether CVRS was associated with progression to dementia with dichotomization as “high CVRS” and “low CVRS” using the maximally selected rank statistics[18]. Multivariate Cox analysis was performed to identify independent determinants of dementia progression with relevant covariates. The retention threshold was set to *p*<0.2 in univariate Cox regression analysis and clinically important measures were also included such as age, sex, educational level, MMSE score, CDR-SOB score, amyloid PET abnormality, ApoEε4 status, and CVRS. Multicollinearity among the covariates was tested by calculating the variance inflation factor[19].

To assess the effect of initial CVRS score on cognitive performance presented by ADAS-cog over time, we fitted the random effect model (with random intercept and slope functions). The random effects model was used because this can account for the correlation that may exist across multiple measurements in the same individual over time[20]. The fixed effects in the model included CVRS, age, educational level, and follow-up time (expressed in months from baseline MRI acquisition). This also included the interaction terms between each variable measured at follow-up visit and CVRS, which was modeled as fixed effects. The intercept and the follow-up time (in months) were included as random effects in the model. All the subjects were assumed to be independent.

The level of statistical significance was set at p<0.05. Statistical analyses were performed using R (Version 3.4.3, The R Foundation for Statistical Computing, 64-bit platform). Cox regression analysis was performed with survival package[21], the optimal cutpoints of continuous variables in the survival analysis was obtained using the maxstat package[18] and panel analysis with the random effect model was performed using the nlme package[22].The graphics were generated using the ggplot2 package[23].

## Results

A total of 340 patients were included in the study. The median age of the patients was 71.3 years, and 159 (46.6%) were female. A total of 156 patients (45.8%) had at least one *APOE* ε4 allele. During the follow-up period (median, 36 months), we observed that 69 patients (20.2%) progressed to dementia, while 271 patients did not. Classification of the demographic, cognitive, and biomarker characteristics based on the progression to dementia as stable MCI and progressive MCI are represented in Table 1. Patients with MCI that progression to dementia had poorer cognitive performances at baseline, higher amyloid PET abnormalities and CVRS scores, and were more likely to be APOE4 carriers than those without progression.

**Table 1:** Baseline characteristics of the patients with MCI.

	Stable MCI (n = 271)	Progressive MCI (n = 69)	Total (n = 340)	*p* value
Age, years (mean ± SD)	71.1 ± 7.5	72.1 ± 7.2	71.3 ± 7.4	0.312
Female, n	127 (46.9%)	32 (46.4%)	159 (46.6%)	1.000
Education, years	16 (14–18)	16 (14–18)	16 (14–18)	0.655
APOE ε4 carriers, n	109 (40.2%)	47 (68.1%)	156 (45.8%)	< 0.001
CDR-SOB	1 (0.5–1.5)	2 (1.5–3.0)	1.5 (0.8–2.0)	< 0.001
ADAS-cog 11	7 (5–10)	12 (9.0–16.0)	9 (6–11)	< 0.001
MMSE	29 (28–30)	28 (26–29)	29 (27–29)	<0.001
Positive amyloid PET, n	128 (47.2%)	61 (88.4%)	189 (55.4%)	< 0.001
CVRS	9 (5–13)	13 (8–17)	10 (6–14)	< 0.001
Hippocampal atrophy	3 (1–4)	4 (3–6)	3 (2–4)	< 0.001
Cortical atrophy	3 (2–5)	5 (3–7)	4 (2–5)	< 0.001
Subcortical atrophy	2 (1–3)	3 (2–4)	2 (1–3)	0.001
Small vessel disease	1 (0–2)	1 (0–2)	1(0–2)	0.729

Values are presented as median ± interquartile range unless otherwise stated. *SD*, Standard deviation, *CDR-SOB* Clinical Dementia Rating Sum of Boxes, *ADAS-cog* Alzheimer’s disease assessment scale-cognitive subscale, *MMSE* Mini-Mental State Examination, *CVRS* Comprehensive Visual Rating Scale.

**Fig 2:**
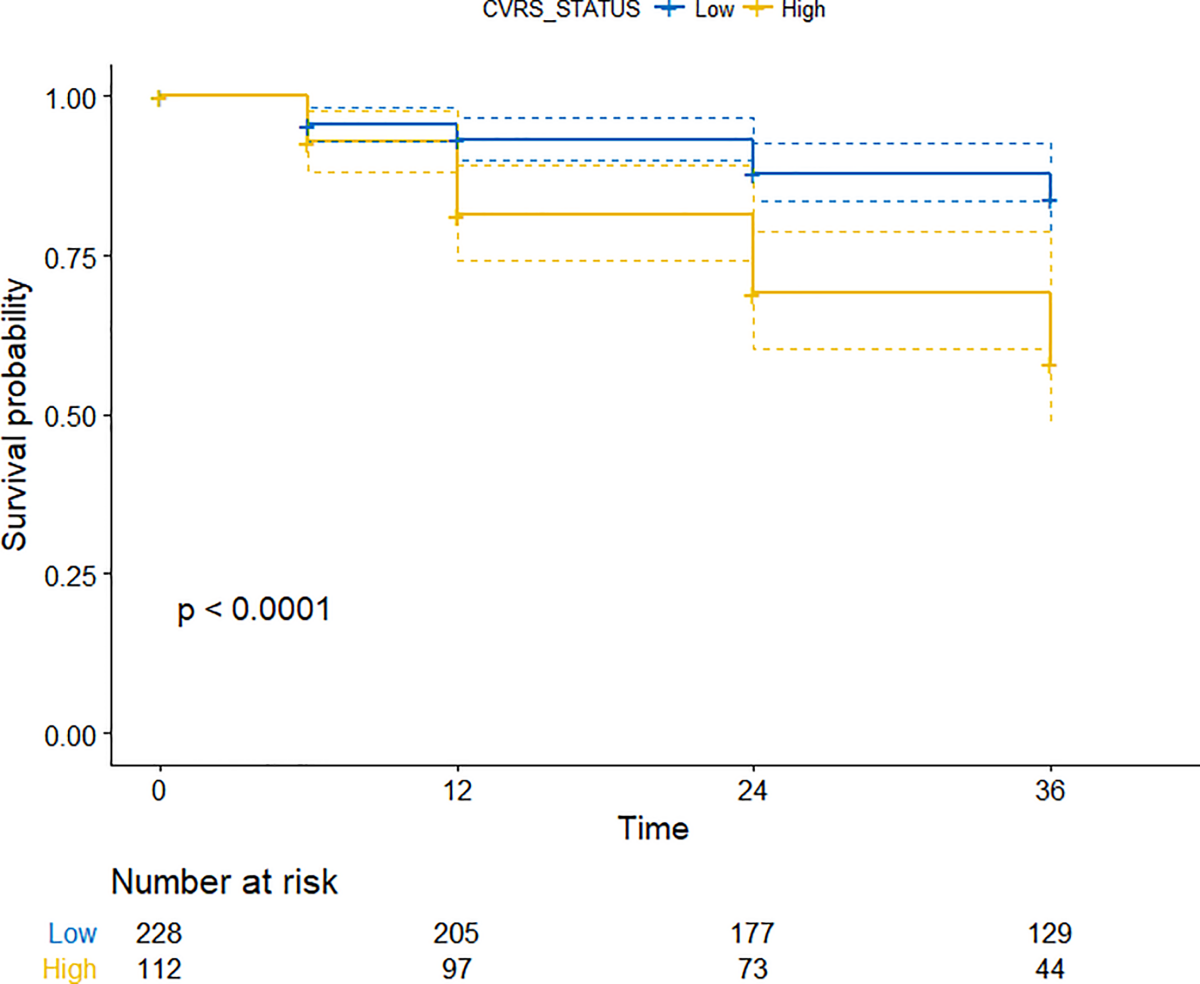
Cox proportional hazards model for progression to dementia in mild cognitive impairment patients according to the CVRS score (‘Low’ indicates ≤12 points, ‘High’ indicates >12 points).

In the univariate Cox regression analysis, patients with higher CVRS scores (>12 points) showed a significantly increased HR (95% CI) of 1.120 (1.070–1.170) for progression to dementia (Fig 2, Table 2). The rate of progression to dementia was significantly higher for APOE ε4 carriers or subjects with amyloid positivity. Baseline cognitive performances with lower MMSE scores, higher CDR Sum of Boxes (CDR-SOB), and higher Alzheimer’s Disease Assessment Scale-cognitive subscale 11 (ADAS-cog 11) scores were also associated with progression to dementia.

**Table 2:** Univariate and multivariate Cox regression analysis.

	Univariate analysis		Multivariate analysis	
	HR(95% Cl)	P-value	HR(95% Cl)	P-value
Age	1.020 (0.990–1.050)	0.226	0.968 (0.926–1.012)	0.152
Male	0.990 (0.620–1.590)	0.970	0.771 (0.448–1.326)	0.347
Education	0.990 (0.900–1.080)	0.821	1.055 (0.956–1.164)	0.286
*APOE* ε4 carrier	2.010 (1.470–2.750)	<0.0001	1.391 (0.930–2.080)	0.108
**Cognition**				
MMSE	0.770 (0.680–0.870)	<0.0001	0.894 (0.771–1.035)	0.134
CDR-SOB	2.380 (1.980–2.870)	<0.0001	2.218 (1.780–2.764)	<0.0001
ADAS-cog 11	1.270 (1.210–1.330)	<0.0001	NI	NI
**Brain imaging**				
Positive amyloid PET	7.140 (3.420–14.930)	<0.0001	4.428 (1.966–9.976)	0.0003
CVRS	1.120 (1.070–1.170)	<0.0001	1.123 (1.059–1.192)	0.0001

*ADAS-cog* Alzheimer’s disease assessment scale-cognitive subscale, *CDR-SOB* Clinical Dementia Rating Sum of Boxes, *CI* confidence interval, *HR* hazard ratio, *MMSE* Mini-Mental State Examination, *CVRS* Comprehensive Visual Rating Scale, *NI* not included

Multivariate Cox analysis included clinically (age, sex, level of education) and statistically relevant variables (*APOE* ε4 allele, MMSE, CDR-SOB, amyloid PET positivity, and CVRS) (Table 2). Although ADAS-cog was statistically relevant and variance inflation factors were <1.550 for all variables, indicating a low degree of collinearity, we excluded ADAS-cog from the multivariate Cox analysis, because ADAS-cog score was clinically highly correlated with MMSE. The adjusted covariates did not alter the significance of the HRs (95% CI) of CVRS [1.123 (1.059–1.192)]. On the contrary, the significant relationships between APOE ε4, initial MMSE, and disease progression were not observed after adjustment with other covariates.

We performed a ROC analysis and estimated area under the curve to assess the diagnostic utility of the CVRS between stable MCI and progressive MCI compared to subscales (Table 3). As described in Table 3, the AUC of the CVRS was greater than that of any other single subscale and volumetric measurement.

**Table 3:** Comparison of area under the curve (AUC) of the CVRS and subscales between stable MCI and progressive MCI.

	Sensitivity, %	Specificity, %	Positive predictive value, %	Negative predictive value, %	AUC	95% CI
CVRS	63.8	65.7	12.3	67.9	0.677	0.605–0.749
Hippocampal atrophy	76.8	49.1	10.7	72.3	0.671	0.601–0.741
Cortical atrophy	58.0	67.9	13.6	68.5	0.662	0.590–0.734
Subcortical atrophy	63.8	59.4	13.4	71.4	0.631	0.558–0.705
Small vessel disease	11.6	92.3	19.8	72.4	0.513	0.440–0.586

*AUC*, area under the curve; *CI*, confidence interval; *CVRS*, Comprehensive Visual Rating Scale; *MCI*, mild cognitive impairment

**Fig 3:**
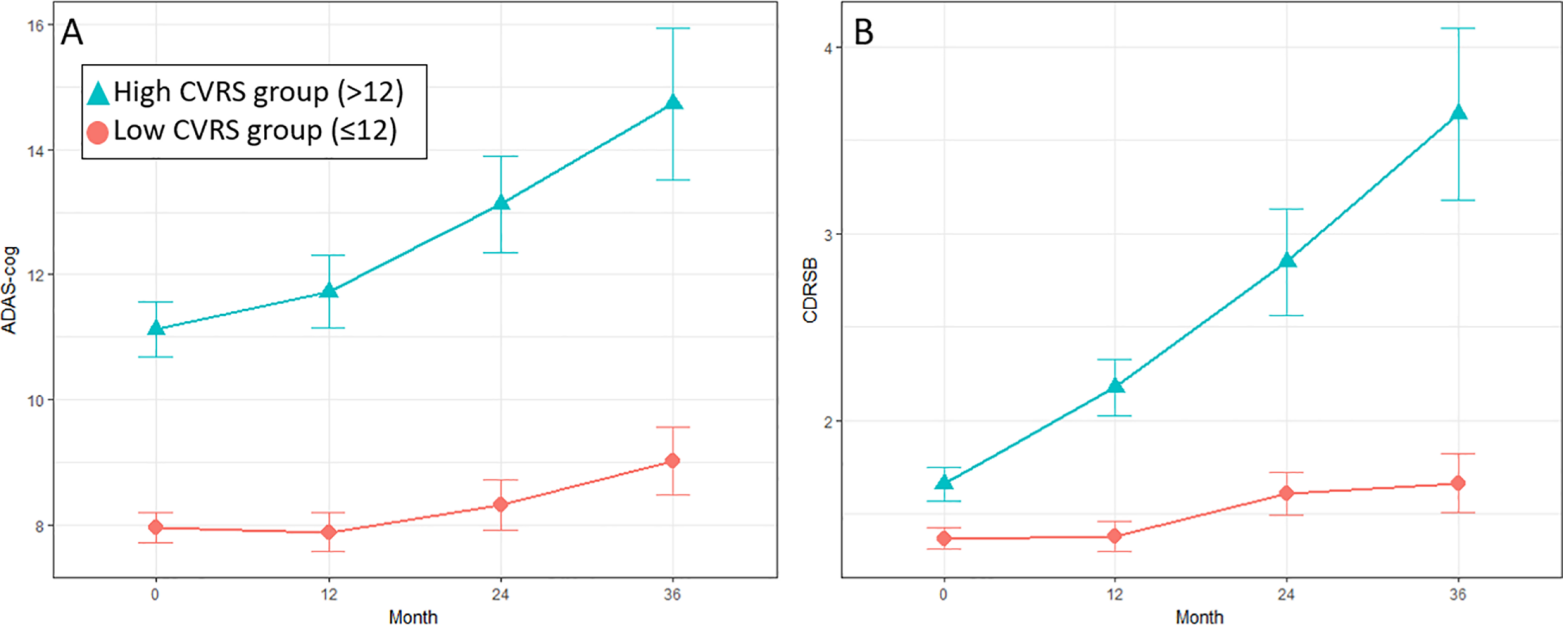
Plot of means with 95% confidence interval of ADAS-cog score (A) and CDR-SOB (B) by CVRS status over time.

Fig 3 summarizes the change in the ADAS-cog11 scores over time according to dichotomized CVRS status. Table 4 represents the parameter estimates, standard error, and *p* value for change in ADAS-cog estimated by the random effects model. Effects on CVRS in terms of annual follow-up visits from the baseline were significant in trajectory of cognitive performance. The effect of interaction between CVRS and the follow-up visit interval on cognitive performance was marginal at 12 months (β estimate = 0.065 units; p<0.075) but became significant after 24 months (β estimate = 0.166 units; p<0.001) and 36 months (β estimate = 0.274 units; p<0.001)

**Table 4:** Results from random effects model: A change in ADAS-cog is associated with variables in MCI patients for 36 months.

Variable (model term)	Estimate (SE)	P Value
Intercept	7.288 (2.737)	0.007
Age at baseline	-0.027 (0.33)	0.416
Education	-0.008 (0.072)	0.897
CVRS	0.359 (0.053)	<0.001
Visit 2 (12 month)	-0.542 (0.406)	0.182
Visit 3 (24 month)	-0.780 (0.541)	0.149
Visit 4 (36 month)	-0.456 (0.834)	0.585
Visit 2 (12 month) x CVRS	0.065 (0.037)	0.075
Visit 3 (24 month) x CVRS	0.166 (0.049)	<0.001
Visit 4 (36 month) x CVRS	0.274 (0.076)	<0.001

*ADAS-cog* Alzheimer’s disease assessment scale-cognitive subscale, *CVRS* Comprehensive Visual Rating Scale.

## Discussion

We investigated the effects of baseline cerebral structural changes determined by CVRS on the progression to dementia and on longitudinal cognitive decline among elderly patients with MCI. The key finding of the study is that MCI patients with higher CVRS at baseline were more likely to progress to dementia during the 3-year follow-up. Additionally, cognitive decline was accelerated by the synergic interaction between the CVRS and follow-up visits.

Although not all individuals with MCI progress to dementia, they are at a higher risk than cognitively normal individuals[24]. If individuals with a high risk of progression to dementia are identified early, preventive intervention can be administered [25]. The CVRS scores for MCI could help identify individuals that are most likely to be referred for additional biomarker studies that are more expensive or invasive, such as PET scanning or CSF analyses. The CVRS scores could also be used in clinical settings without the need for additional high-end biomarkers except for brain MRI.

Longitudinal studies provide important insights into the synergistic interaction between the CVRS and follow-up visits in cognitive decline (Table 4). Although CVRS affected cognitive decline, the effects of synergic interaction increased with each follow-up visit and attained statistical significance after 24 months. The subtotal scores of the cerebral atrophy scales were 23 points and those of small vessel disease were 7 points, which totaled 30 points for the CVRS. As approximately three-quarters of the CVRS consist of atrophy scores, it mainly reflects cerebral atrophy on MRI, which is a biomarker of neurodegeneration or neuronal injury. This is classified as ‘N group’ according to new A/T/N classification[26] and it is regarded as a non-specific marker that can be observed in wide variety of pathologic conditions including AD, cerebrovascular disease, epilepsy, anoxia, hippocampal sclerosis, TDP-43-opathy, primary age-related tauopathy, chronic traumatic encephalopathy, argyrophilic grain disease, and non-AD primary tauopathies [6,7,26–28]. As for AD, neurodegeneration might be the final result of the β-amyloid plaque or associated pathologic state (labeled as ‘A’) and subsequent aggregated pathologic tau (labeled as ‘T’). Therefore, our findings of increased slope of cognitive decline between 12 months and 24 months according to CVRS status (Fig 2) and increasing effects with follow-up visit after 24 months might be explained by the temporal evolution of biomarkers implying that marker ‘N’ represents disease progression, which is altered at a relatively later stage of the biomarker cascade[29–31].

About a quarter of the total scores of the CVRS consists of scores for small vessel disease (7/30 points), which includes vascular injury markers, such as white matter hyperintensities, lacunar infarcts, and microbleeds. Mixed pathologies, such as the coexistence of neurodegenerative and cerebrovascular disease are increasingly recognized as important for AD and other forms of dementia by longitudinal clinical-pathological studies[10]. Updated data from the religious orders study and rush memory and aging project showed almost 75% of individuals with a pathologic diagnosis have one or more of the vascular pathologies[32,33]. Vascular pathology is present in about 90% of individuals with probable AD and mixed AD pathology, and other degenerative diseases in approximately 65%. In over 58% of individuals, MCI has been observed in combination with vascular pathologies such as microinfarcts, atherosclerosis, arteriolosclerosis and cerebral amyloid angiopathy. While some studies suggest that vascular pathologies directly increase the likelihood of clinical AD, others suggest that there is a synergistic interaction between AD and vascular pathologies[34]. The contribution of vascular pathologies to other pathologies and exact mechanism of vascular cognitive impairment remains an area that requires to be studied.

Although automated analyses of cerebral structural change or vascular injury have already been developed and are being used widely in research field, visual rating involving scales such as the CVRS is easier and quicker, and does not generally require specific MRI or software; it is more suitable for individual assessment in a clinical setting[12,35–37]. However, this does not imply that the CVRS is generally better than automated measurement because atrophy measured by the CVRS was not adjusted for total intracranial volume; hence, the objectivity is relatively poorer. Nevertheless, it is a cost-effective diagnostic tool that is ideally suited for implementation in clinical practice[38]. A visual rating scale, such as CVRS better reflects the observations of a clinician on brain MRI and is a simple score that might be useful for assessing an individual in a primary clinical setting. In contrast, automated imaging analysis tools are more appropriate for detailed research with group analyses in a longitudinal follow-up [39].

Several MRI visual rating scales have already been developed to assess a variety of brain lesions[37,40–43]. Some of them, such as Scheltens’ medial temporal atrophy scale[44] are the most successful as it was used extensively studies, clinical trials, and has been recommended in the diagnostic guidelines of AD[45], while other scales have little or no impact without subsequent replication[38]. Our study was in line with previous studies, in which visual rating scales that focused on validation, correlated with clinical measures of cognition in a clinically relevant population[12]. However, CVRS has its own strengths and novelty as it suggests unified integration of other validated scales based on neurodegeneration and vascular injury while others have only investigated a single scale dependent on a specific diagnosis such as AD, frontotemporal dementia or vascular dementia[38]. Furthermore, we provided an intuitive template for visual rating either using a tablet-computer or a table demarcated with a bounding box for region of interest to be clear at rating procedure (S1 Fig and S2 File).

Our study has several limitations. First, the score of small vessel disease revealed no difference between stable MCI and progressive MCI (Table 2) and the prevalence of lacunes and microbleeds in our patients was low (lacunes, 7.9%; microbleeds, 5.3%), compared with the findings of previous studies[46–48]. ADNI included individuals with Hachinski scores ≤ 4 and excluded those with multiple lacunes; hence, the effects of small vessel disease might be underestimated in the current study. Considering that vascular damage with a white matter hyperintensity or lacune is known to be associated with increased brain atrophy in the context of AD pathology in the pre-dementia stage[49,50] or worse cognitive outcome [51], the effects of the subscales of small vessel disease in CVRS could be the target of validation in the future research. Second, although a change in slope, which indicates the rate of deterioration of ADAS-cog and CDR-SOB, was observed between 12 months and 24 months, cognition was not measured intermediately; hence, impossible to indicate the important inflection points (Fig 3). Third, we included MCI subjects who performed both MRI and amyloid PET at baseline, that might result in selection bias. Although, amyloid PET is not part of the current standard care, a meta-analysis of studies evaluating amyloid PET’s ability to predict MCI conversion to AD demonstrate a sensitivity of 93% and a specificity of 56%[52]. Considering growing importance of amyloid PET in clinical practice, we included it as one of the selection criteria to confirm CVRS as independent predictor. Lastly, it is probable that the weighed subscale value will better reflect the influence of the effects of each subscale on global cognition. However, this weighted method has not been adopted, because it can ruin the simplicity of the CVRS by converting the output of the scores from integers to real numbers. Furthermore, it did not have significant superiority over the non-weighted method for group discrimination and correlation with cognitive function[12].

In conclusion, this study showed that initial CVRS scoring in an individual with MCI is independently associated with disease progression to dementia over a 3-year follow-up period. Moreover, cognitive decline was accelerated by the synergic interaction between the CVRS and follow-up visits. This indicates that CVRS can be used to predict disease progression in patients with MCI.

## Supporting information

S1 Fig Flowchart of the scoring of the comprehensive visual rating scale (CVRS). (TIF)

S1 File The full description of how the comprehensive visual rating scale (CVRS) score is calculated. (DOCX)

S2 File The comprehensive visual rating scale (CVRS) on a tablet computer. (DOCX)

S1 Table Values for inter-rater and intra-rater reliability of CVRS and subscales. (DOCX)
