# Supplementary figures and images for: A ‘Comprehensive Visual Rating Scale’ for predicting progression to dementia in patients with mild cognitive impairment
Source: PLoS One. 2018 Aug 20;13(8):e0201852. doi: 10.1371/journal.pone.0201852 (PMC6101367; doi:10.1371/journal.pone.0201852)

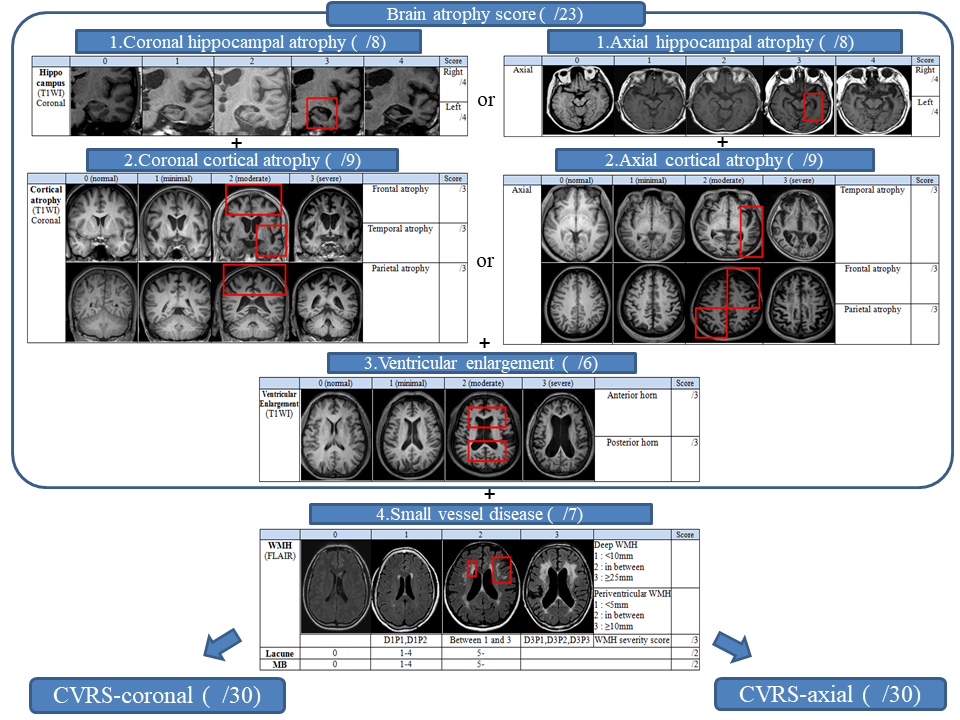

Supplement: S1 Fig — (TIF) [file pone.0201852.s001.tif]
