# Supplementary material for: A ‘Comprehensive Visual Rating Scale’ for predicting progression to dementia in patients with mild cognitive impairment
Source: PLoS One. 2018 Aug 20;13(8):e0201852. doi: 10.1371/journal.pone.0201852 (PMC6101367; doi:10.1371/journal.pone.0201852)
Supplement: S1 File — (DOCX) [file pone.0201852.s002.docx]

**S1 File.** The full description of how the Comprehensive Visual Rating Scale (CVRS) score is calculated

1. **Hippocampal atrophy**

Hippocampal atrophy was measured on coronal T1-weighted images with Scheltens’ scale [1] that is based on the surrounding cerebrospinal fluid space and the hippocampal height in the left and right hemispheres. A coronal template image is a slice that shows the cerebral peduncle most prominently.

1. **Cortical atrophy**

Cortical atrophy was determined by rating the axial images and coronal images separately with 8 template images and a four-point scale (0, 1, 2, and 3) that was modified from Victoroff’s visual rating scale[2]. The original Victoroff’s method used 6 standard T1-weighted images and a four-point scale (0, 0.5, 1, and 2) that measures the anterior frontal lobe with axial images and anterior temporal and midparietal lobes with coronal images. However, we modified this because of the complexity of the use of axial and coronal images at the same time. Our coronal template images included slices that showed both temporal stems connecting the temporal and frontal lobes to assess frontal and temporal atrophy and a slice posterior to the splenium of the corpus callosum to assess parietal atrophy. The axial template images included slices that showed the superior colliculus to assess temporal atrophy and the first slice above the lateral ventricle to assess frontal and parietal atrophy. The parietal atrophy assessment was very similar to the posterior cortical atrophy (PCA) scale by Koedam et al[3], although the CVRS was simpler because it did not include a regional index (posterior cingulate sulcus and the parieto-occipital sulcus) or sagittal images. More severe atrophy was used to evaluate when there was asymmetry.

1. **Ventricular enlargement as a representation of subcortical atrophy**

We measured ventricular enlargement on the T1-weighted images with a template-based 4-point scale (0, 1, 2, and 3) by examining the enlargement of the anterior and posterior lateral ventricles separately, which was a modification of a previously published method[4]. The anterior and posterior horns of the lateral ventricle were rated separately, which was adequate because there were many cases with anterior and posterior discrepancies of ventricular size. The use of template-based ventricular enlargement as a representation of subcortical atrophy has also been used in a previous study by the LADIS group who showed good correlations with cognitive decline [5].

1. **White matter hyperintensity (WMH)**

The severity of WMHs was evaluated according to the modified Fazekas and Scheltens scale on T2 axial FLAIR images [6]. WMHs were rated in the periventricular white matter (PWM, P rating) and deep white matter (DWM, D rating) areas separately, and the D and P ratings were combined to provide a final ischemia score. DWM lesions were divided into D1 (DWM < 10 mm), D2 (10 ≤ DWM < 25 mm), and D3 (≥25 mm) based on the longest diameter of the lesions. PWM lesions were classified into P1 (cap and band < 5 mm), P2 (between P1 and P3), and P3 (cap or band ≥ 10 mm) based on the size of the cap and band, which were perpendicular and horizontal to the ventricle, respectively. The results were combined to provide a representative rating of minimal (D1P1 or D1P2), moderate (between the minimal and severe group), or severe (D3P1, D3P2, or D3P3). Finally, the group with no WMHs was rated 0, the minimal group was 1, the moderate group was 2, and the severe group was 3[7,8].

1. **Lacunes and microbleeds**

Lacunes were defined as cavities with a size of 3 to 10 mm with signal intensities that were similar to CSF on FLAIR, T1, and T2 images to distinguish lacunes from microbleeds and Virchow Robin spaces[9]. The number of lacunes was recorded as grade 0 (no lacunes), grade 1 (1–4 lacunes), or grade 2 (5 or more lacunes), which was also used in a previous study[10]. Microbleeds were defined as focal areas with very low signal intensities on gradient-recalled echo images. Signal voids by sulcal vessels, symmetrical calcification in the basal ganglia, the choroid plexus, and pineal calcification were excluded[11]. The number of microbleeds was graded as grade 0 (no microbleeds), grade 1 (1–4 microbleeds), or grade 2 (5 or more microbleeds) based on the Rotterdam Scan Study of the association between cerebral microbleeds and performance in multiple cognitive domains[12].

1. Scheltens P, Leys D, Barkhof F, Huglo D, Weinstein HC, Vermersch P, et al. Atrophy of medial temporal lobes on MRI in “probable” Alzheimer’s disease and normal ageing: diagnostic value and neuropsychological correlates. J Neurol Neurosurg Psychiatry. 1992;55: 967–972.

2. Victoroff J, Mack WJ, Grafton ST, Schreiber SS, Chui HC. A method to improve interrater reliability of visual inspection of brain MRI scans in dementia. Neurology. 1994;44: 2267–2276.

3. Koedam ELGE, Lehmann M, Flier WM van der, Scheltens P, Pijnenburg YAL, Fox N, et al. Visual assessment of posterior atrophy development of a MRI rating scale. Eur Radiol. 2011;21: 2618. doi:10.1007/s00330-011-2205-4

4. O&#39 J, Donovan, Watson R, Colloby SJ, Firbank MJ, Burton EJ, et al. Does posterior cortical atrophy on MRI discriminate between Alzheimer’s disease, dementia with Lewy bodies, and normal aging? Int Psychogeriatr. 2013;25: 111–119. doi:10.1017/S1041610212001214

5. Jokinen H, Lipsanen J, Schmidt R, Fazekas F, Gouw AA, van der Flier WM, et al. Brain atrophy accelerates cognitive decline in cerebral small vessel disease: The LADIS study. Neurology. 2012;78: 1785–1792. doi:10.1212/WNL.0b013e3182583070

6. Scheltens P, Erkinjunti T, Leys D, Wahlund L-O, Inzitari D, Ser T del, et al. White Matter Changes on CT and MRI: An Overview of Visual Rating Scales. Eur Neurol. 1998;39: 80–89. doi:10.1159/000007921

7. Noh Y, Lee Y, Seo SW, Jeong JH, Choi SH, Back JH, et al. A new classification system for ischemia using a combination of deep and periventricular white matter hyperintensities. J Stroke Cerebrovasc Dis Off J Natl Stroke Assoc. 2014;23: 636–642. doi:10.1016/j.jstrokecerebrovasdis.2013.06.002

8. Park HK, Na DL, Han S-H, Kim J-Y, Cheong H-K, Kim SY, et al. Clinical Characteristics of a Nationwide Hospital-based Registry of Mild-to-Moderate Alzheimer’s Disease Patients in Korea: A CREDOS (Clinical Research Center for Dementia of South Korea) Study. J Korean Med Sci. 2011;26: 1219–1226. doi:10.3346/jkms.2011.26.9.1219

9. Gouw AA, van der Flier WM, Fazekas F, van Straaten ECW, Pantoni L, Poggesi A, et al. Progression of white matter hyperintensities and incidence of new lacunes over a 3-year period: the Leukoaraiosis and Disability study. Stroke. 2008;39: 1414–1420. doi:10.1161/STROKEAHA.107.498535

10. Benisty S, Gouw AA, Porcher R, Madureira S, Hernandez K, Poggesi A, et al. Location of lacunar infarcts correlates with cognition in a sample of non-disabled subjects with age-related white-matter changes: the LADIS study. J Neurol Neurosurg Psychiatry. 2009;80: 478–483. doi:10.1136/jnnp.2008.160440

11. Greenberg SM, Vernooij MW, Cordonnier C, Viswanathan A, Al-Shahi Salman R, Warach S, et al. Cerebral microbleeds: a guide to detection and interpretation. Lancet Neurol. 2009;8: 165–174. doi:10.1016/S1474-4422(09)70013-4

12. Poels MMF, Ikram MA, van der Lugt A, Hofman A, Niessen WJ, Krestin GP, et al. Cerebral microbleeds are associated with worse cognitive function: the Rotterdam Scan Study. Neurology. 2012;78: 326–333. doi:10.1212/WNL.0b013e3182452928
