# Supplementary material for: A ‘Comprehensive Visual Rating Scale’ for predicting progression to dementia in patients with mild cognitive impairment
Source: PLoS One. 2018 Aug 20;13(8):e0201852. doi: 10.1371/journal.pone.0201852 (PMC6101367; doi:10.1371/journal.pone.0201852)

**S2 File.** The Comprehensive Visual Rating Scale (CVRS) on a tablet computer (the iPAD version)


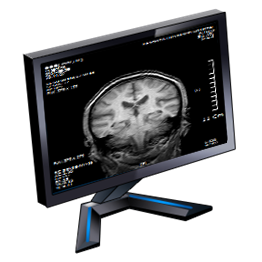


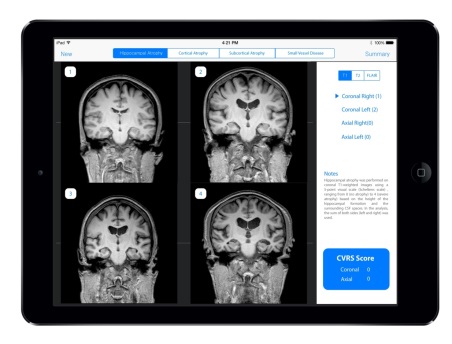


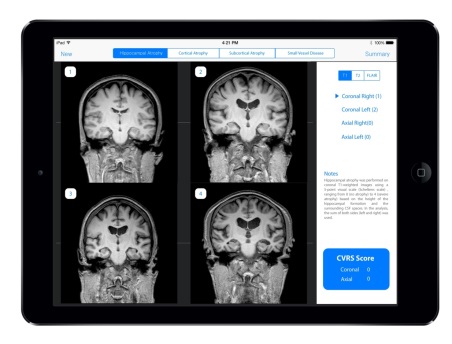

The raters used a template-based scoring program on a tablet computer that summed the total score automatically by matching the closest template image to the real magnetic resonance imaging (MRI) finding of the subject.

Comparing actual brain MRI of the patient and template image of IPAD, raters can select the most similar template image on the iPad and touch it to save the score on the iPad. In this way, if you touch the template images related to cortical atrophy, hippocampal atrophy, ventricular enlargement, and small vessel disease one by one, the scores will be combined and the total score and each partial score will be output on the last screen.

The template of CVRS was made based on reference plane with anterior commissure-posterior commissure (AC-PC) line for axial image. Because this has been adopted as standard for routine brain MR scan, we thought that there will be no significant problem for the visual rating of routine MR in clinical practice. However, in the case of 3D-T1 images, it may look different from the template images and it is necessary to align the orientation to template image using image viewing software such as MRIcro or mricron.

The main screenshot of this software is as follows.


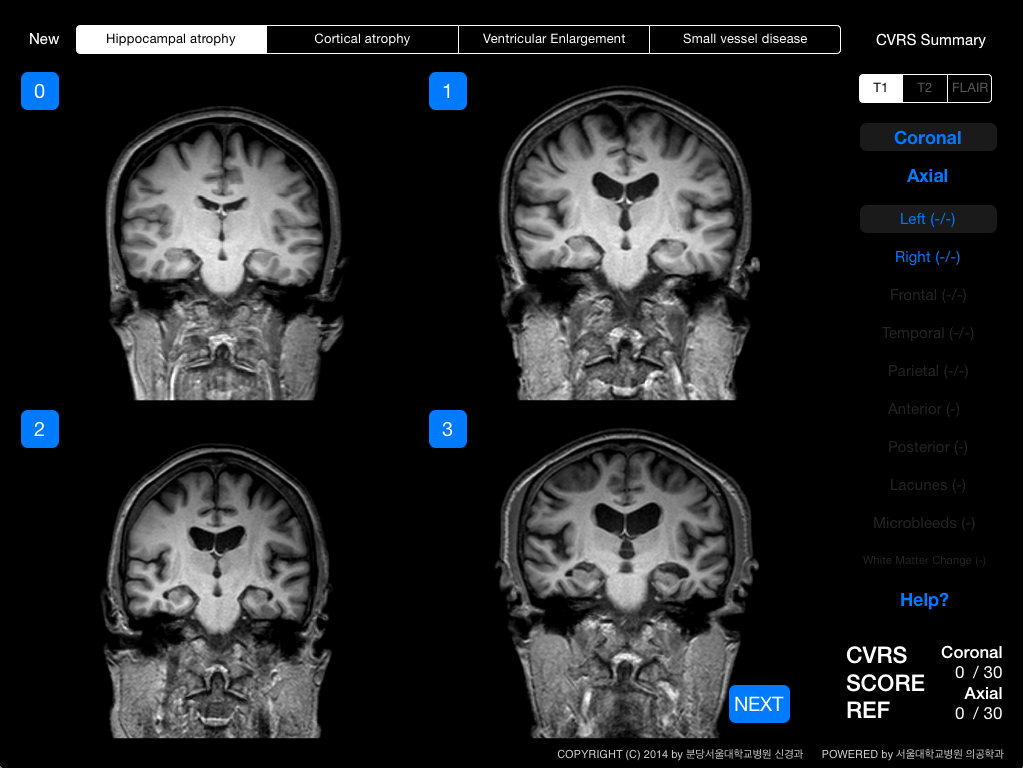


The upper menu bar indicates the 4 subscales (hippocampal atrophy, cortical atrophy, ventricular enlargement, and small vessel disease). The user can move to each of them by touching it. The right menu bar shows the summed score as well as the imaging modality. Although the CVRS is based on intuitive visual ratings, help buttons show the principles of each visual rating described in the methods section. Detailed screenshots of the CVRS follow.

1.
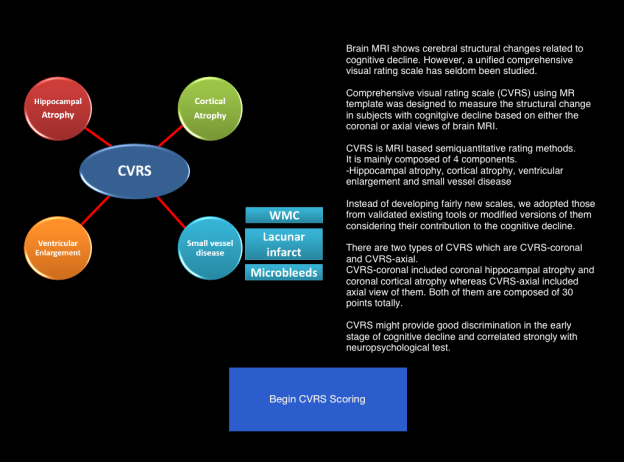
The first screenshot for the CVRS. It includes the introduction. The user can move to the scoring section by touching the blue button labeled *Begin CVRS Scoring* at the bottom of the screen.

2. Hippocampal atrophy (coronal or axial views). By matching and touching the closest template image to the real MRI finding of the subject, the score for coronal or axial hippocampal atrophy is automatically calculated in the right menu bar. The user can move to the grade-4 image by touching the *next* button at the right lower side of the screen. Left and right hippocampal atrophy are measured separately by using the buttons labeled *left* and *right* in the right menu bar.


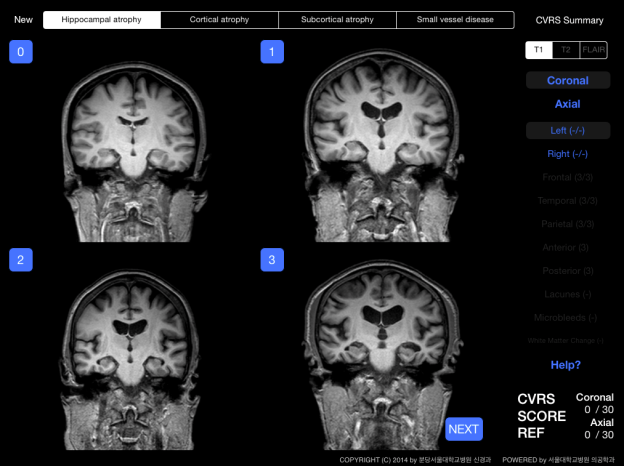

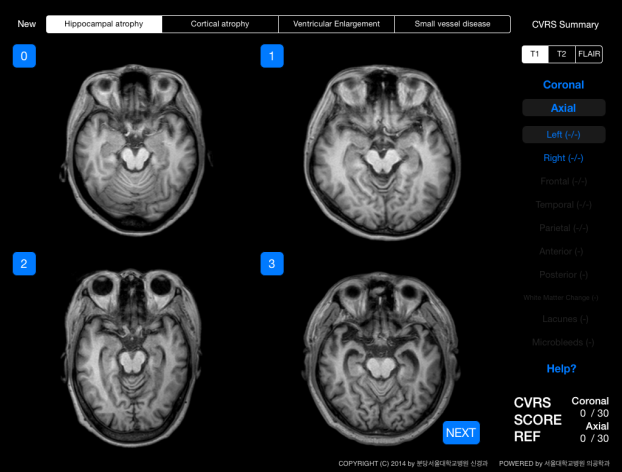


3. Cortical atrophy ( coronal or axial views). Frontal, temporal, and parietal atrophy can be rated. The user can move to the image of each lobe by touching the buttons labeled *Frontal*, *Temporal*, or *Parietal* in the right menu bar. The ratings are done for the three lobes for the coronal or axial views.


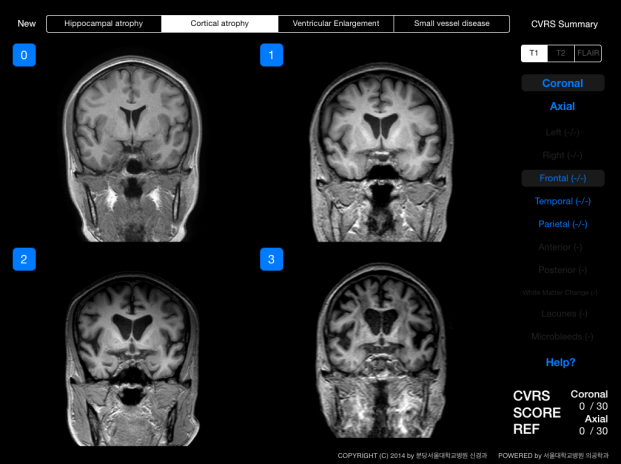

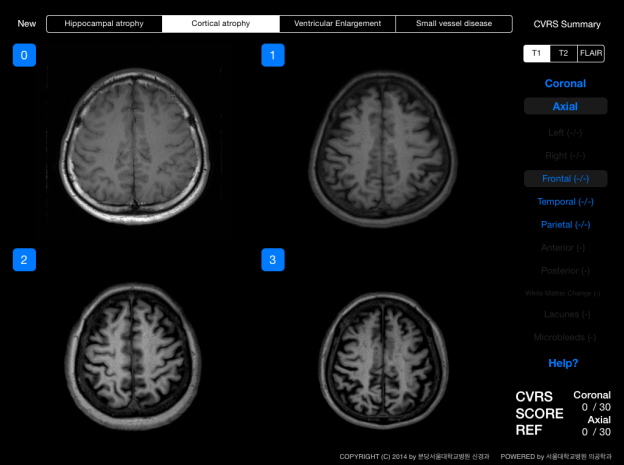


4. Ventricular enlargement. Ventricular enlargement was measured by rating the enlargement of the anterior and posterior lateral ventricles separately (Left figure).

5. The white matter change (WMC). WMC was measured according to the modified Fazekas and Scheltens scale (Right figure).


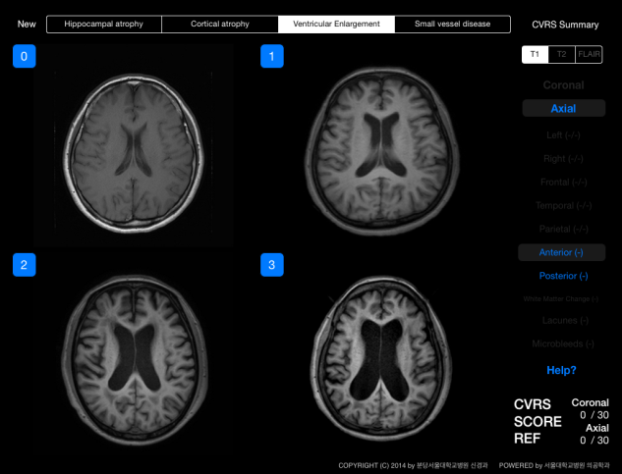

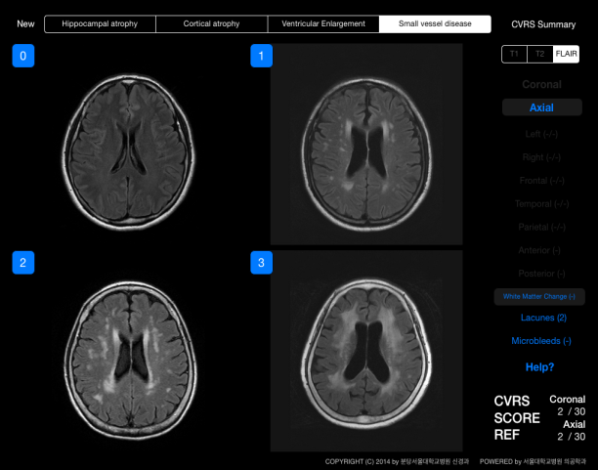


6. The number of lacunes and microbleeds are entered, and their scores are automatically calculated as grade 0 (no lesions), grade 1 (1–4 lesions), or grade 2 (5 or more lesions).


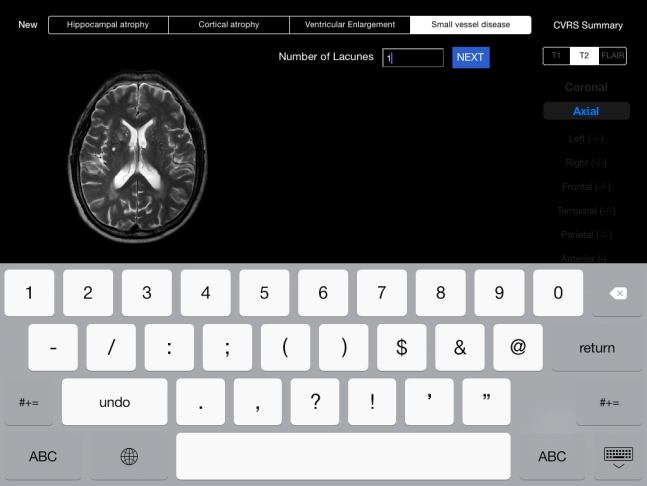

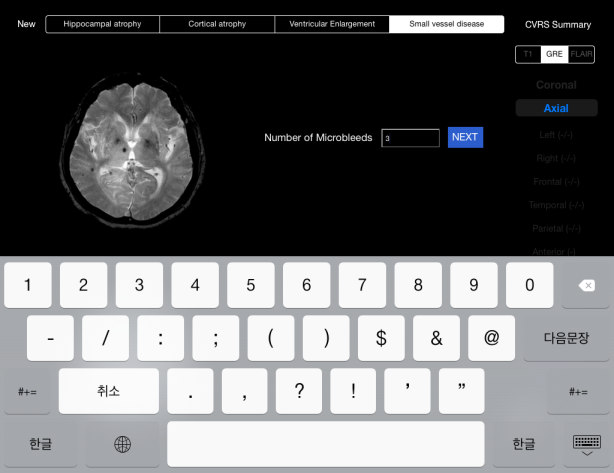


7. The summary of the scores. The summary shows the total summed score of the CVRS as well as the subscores of the subscales (left figure). Personal information can be entered for the subjects, and all of the data can be transferred to a server or individual E-mail (right figure).


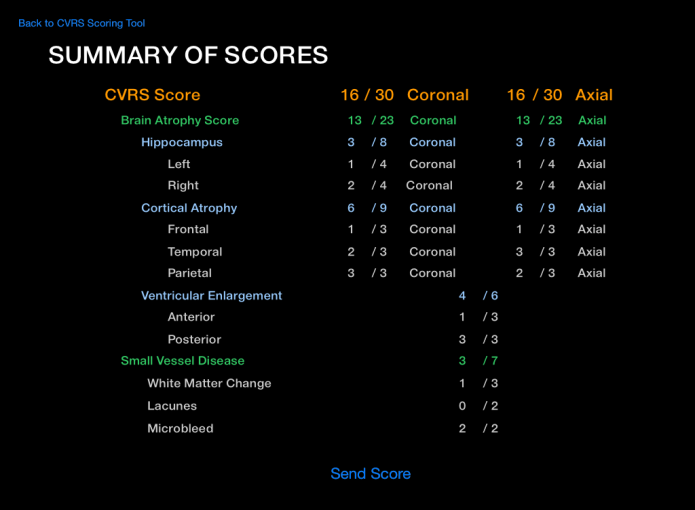

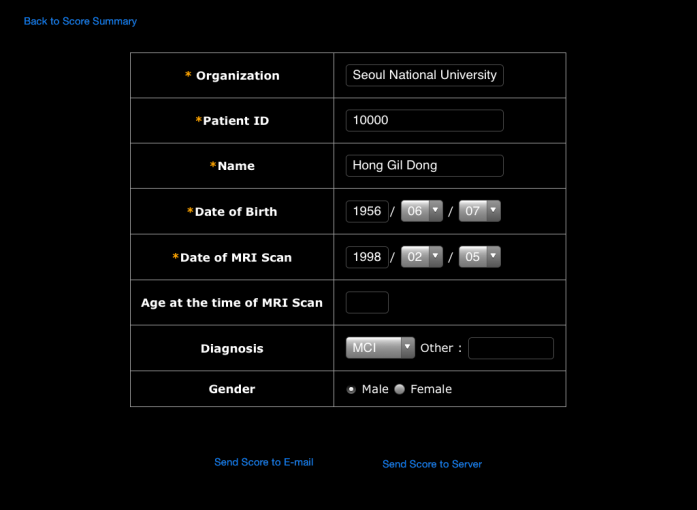

Supplement: S2 File — (DOCX) [file pone.0201852.s003.docx]
