# Supplementary material for: A ‘Comprehensive Visual Rating Scale’ for predicting progression to dementia in patients with mild cognitive impairment
Source: PLoS One. 2018 Aug 20;13(8):e0201852. doi: 10.1371/journal.pone.0201852 (PMC6101367; doi:10.1371/journal.pone.0201852)
Supplement: S1 Table — (DOCX) [file pone.0201852.s004.docx]

**Table S1.** Values for inter-rater and intra-rater reliability of CVRS and subscales

|  | Inter-rater (95% CI) | Intra-rater (95% CI) |
| --- | --- | --- |
| CVRS | 0.941 (0.902-0.965) | 0.936 (0.889-0.966) |
| Hippocampal atrophy | 0.864 (0.785-0.920) | 0.888 (0.803-0.938) |
| Cortical atrophy | 0.904 (0.847-0.944) | 0.942 (0.899-0.969) |
| Ventricular enlargement | 0.894 (0.831-0.938) | 0.876 (0.782-0.931) |
| Small vessel disease | 0.903 (0.846-0.944) | 0.901 (0.826-0.945) |

Values are presented as the ICC (95% CI).

*CVRS* Comprehensive Visual Rating Scale, *CI* confidence interval, *ICC* intraclass correlation coefficient
